# Assessing spider fear: Validity of a novel computerized behavioral avoidance test

**DOI:** 10.1371/journal.pone.0346624

**Published:** 2026-05-18

**Authors:** Alexander Karner, Cindy Sumaly Lor, Mengfan Zhang, Frank Scharnowski

**Affiliations:** Department of Cognition, Emotion, and Methods in Psychology, University of Vienna, Vienna, Austria; National Museums of Kenya, KENYA

## Abstract

Spider phobia is one of the most common specific phobias and can be highly detrimental to one’s quality of life. To evaluate the efficacy of treatments, reliable pre- and post-treatment assessments are needed. Behavioral avoidance tests (BATs), where one is asked to approach a spider, are commonly used as such measures. With the increasing popularity of computerized interventions, there is a demand for quick assessments for fear of spiders, that can be completed using computers or smartphones. Here, we tested the validity of a novel computerized BAT (cBAT) that we introduced in our previous work. In this cBAT, participants unblurred three images of spiders up to a point where they still felt comfortable viewing them. We expected that the cBAT would be moderately to strongly correlated with established measures for fear of spiders. Our analysis revealed that the cBAT showed moderate convergent and concurrent validity through correlations with self-report questionnaires indicating spider fear, and with a real-life BAT, respectively. However, the latter was no longer observed in a follow-up. While the cBAT facilitates ease of application and has a quick, straightforward participant task, we conclude that the cBAT is a limited measure for fear of spiders and might not fully capture the complexity of spider-related avoidance behavior compared to tasks that simulate real-life scenarios more closely. We propose the development of a refined cBAT that maintains an easily applicable participant task.

## Introduction

Spider phobia, which is defined by excessive and exaggerated fear towards spiders [1], can be highly detrimental to one’s quality of life and has a prevalence that varies between 2.7 and 9.5% [2–4]. The current gold standard for the treatment of spider phobia is exposure therapy (ET) [5–7], with even single-session treatments showing efficacy [8,9]. Although exposure therapy is highly beneficial, it has a dropout rate of up to 45% [10], and relapses of fear are common [10,11]. Moreover, the availability of therapists is limited, stigma can cause reluctance to undergo treatment, and therapy is often associated with high cost [12]. As alternatives to conventional exposure therapy, a variety of novel treatment approaches have been developed. For instance, virtual reality (VR) exposure therapy [13] has shown some promising results with regard to the treatment of fear of spiders [14–16]. As an even more easily accessible approach, online exposure treatments have been introduced, which can be utilized via the internet [17–19] or through smartphone apps [19–21]. Such computerized applications have the potential to offer treatment to affected individuals that may not enroll in or drop out of conventional exposure therapy. Indeed, several studies have shown that self-exposure to virtual or digital spiders can result in a reduction in fear [17,20,22].

For exposure-based treatments to be successful, it is crucial to assess an individual’s level of fear of spiders, as the intensity of exposure should neither be too overwhelming, nor too easy for an individual [23,24]. Additionally, in order to assess the effectiveness of a treatment, reliable outcome measures are needed. A variety of established tools are available for the detection and the assessment of spider fear. Questionnaires are easily applicable and widespread tools. The Spider Questionnaire [25] is a prominent example with high validity, consisting of true or false question items related to fear of spiders. The Spider Phobia Questionnaire (SPQ) [26] is another widely used questionnaire, also requiring dichotomous responses, but additionally incorporating different domains of spider phobia. Subsequently, the Fear of Spiders Questionnaire [27] was developed, employing question items requiring answers on a 7-point Likert scale, and potentially providing a more fine-grained differentiation between different levels of fear of spiders, particularly in the non-phobic range [28]. Both the FSQ and SPQ have been translated to German and validated [29]. While self-report questionnaires are effective tools for assessing pre-post treatment differences, it has been argued that they may not fully reflect the complexity of an individual’s experience [30]. Besides questionnaires, behavioral avoidance tests [9], where participants are asked to approach a spider, can be used to assess fear levels before and after treatment, for instance, as in a study by Miloff et al. [31]. BATs typically show strong correlations with self-report questionnaires [30,32]. Nonetheless, given the direct contact with the feared stimulus, BATs arguably have a higher ecological validity than self-report questionnaires, providing a more accurate reflection of actual fear reactions [30]. Conventionally, BATs have been conducted in real life [9,32,33], which requires resources, time and planning. However, hand in hand with the development of computerized exposure, computerized BATs have been introduced that could simplify the assessment of fear of spiders before and after an intervention. For instance, Binder et al. [34] developed a VR assessment for spider-related avoidance behavior, in which participants completed three different avoidance tasks using VR glasses. In one of the tasks, participants were instructed to walk towards a table with a spider and touch the animal. Binder et al. [34] found that this task clearly differentiated between phobic, fearful, and non-fearful individuals and displayed strong correlations with FSQ scores. Moreover, phobic individuals needed significantly more time until they touched the spider than fearful or non-fearful individuals. The latter is in line with the results of Frumento et al. [35], who also conducted a VR BAT, and where the time needed to approach the stimulus (but not the distance walked towards the feared object) was predictive of spider fear. While VR BATs are useful, they still require dedicated equipment and are less familiar, making them more complex to implement. Thus, given the general demand for quick assessments for spider phobia [36], computerized interventions could particularly benefit from a fast, reliable assessment that can also be completed online by simply using the internet and a standard computer.

Meng et al. [37] introduced two computer-delivered BATs for spider phobia using pictures and video clips, respectively. Although their results showed good convergent validity (i.e., relations with other measures that measure the same or a similar construct) [38,39], with a real-life BAT (rl-BAT), the computer-delivered BATs failed to discriminate between phobic and non-phobic individuals. The authors concluded that their computer-delivered BATs did not pose a suitable alternative to a rl-BAT. Within the scope of an online exposure game for spider-fearful individuals, Dibbets and Schruers [17] introduced a digital BAT, where participants were presented with an image of a spider in the desert and had to indicate via a mouse click up to which point they would dare to approach it if they encountered it. The authors found moderate correlations of the digital BAT with FSQ scores. Moreover, Grill and colleagues [40] presented a novel computer-delivered online BAT for fear of spiders, with an initial validation showing promising results. Specifically, 31 spider-fearful, as well as 31 non-spider-fearful participants completed the online BAT alongside a rl-BAT. The online BAT was conducted in the style of a first-person video game and started with an imagination exercise that included instructions and lasted for approximately 5 minutes. Then, participants controlled the movements of a virtual character on a computer screen, with the aim to achieve a maximum number of steps in approaching a spider. Among others, these steps included entering an experimental room, where a spider was positioned in a glass box on a desk, approaching the box, and putting a virtual hand inside the box to handle the spider [40]. The rl-BAT, which was adapted from Öst et al. [9], also followed this protocol. In summary, Grill et al. [40] found that their online BAT was highly correlated with the rl-BAT, as well as with self-report questionnaires, and that the online BAT indicated spider-related avoidance equal to the rl-BAT. While the results of Grill et al. [40] are encouraging, their computer-delivered BAT requires substantial time, familiarization and effort from the participants. Thus, it is worth pursuing the development of quick assessments that can be completed in minimal time and with minimal effort or instructions and might also be suitable for apps and remote use. Dibbets and Schruers [17] developed such a quick assessment. However, this task was not validated using a real-life measure such as a BAT, and relied on the imagination of participants, as they had to translate their level of comfort into a hypothetical approach distance.

In a recent data article, we introduced a novel computerized behavioral avoidance test (cBAT) for fear of spiders, which was completed by spider-fearful participants within the scope of a neuroimaging study [41]. This cBAT was designed to facilitate ease of use, has a straightforward participant task and can be completed in approximately 30 seconds, after reading a short instruction text and completing one practice trial. In the cBAT, participants are instructed to unblur three highly blurred images of spiders on a computer screen up to a point where they still feel comfortable viewing them, resulting in a mean unblur value (i.e., cBAT score per participant). This task allows a gradual direct exposure, with stimulus intensity (and consequently discomfort) becoming greater in real-time, without relying on spatial imagination. The cBAT’s rapid and easy application make it a potentially interesting measure for computerized and internet-based applications. In addition to the cBAT, the participants underwent a rl-BAT in which they approached a terrarium with a real spider and filled out self-report questionnaires indicating their level of fear of spiders, as well as questionnaires measuring different constructs. Although the cBAT and the respective data were made publicly available in our previous publication, no statistical analysis of this data was conducted at the time. Here, we employ this data for the first time to examine our novel cBAT as a tool for the assessment of fear of spiders, with the aim to investigate if a reduced, image-based task can deliver valid results. Specifically, we examined the cBAT’s validity in terms of associations with self-report questionnaire sum scores and rl-BAT data. We hypothesized (1) that our cBAT would be moderately to strongly correlated with established instruments measuring fear of spiders (i.e., rl-BAT data and self-report questionnaires) [40], (2) exhibit lower or non-significant correlations with measures of general anxiety, and (3) that higher levels of spider fear would be associated with more time needed to unblur the images in the cBAT [34,35].

## Materials and methods

In the present study, we employed a subset of the data provided in our previous work [41], to assess the cBAT as a measure for fear of spiders. Specifically, we used self-report questionnaire sum scores, cBAT data, as well as rl-BAT data. In the following sections, we provide a summary of the methods of our original publication [41] with relevance to the present article.

### Participants

Participants in our original study [41] were 51 German-speaking adults (mean age = 22.65 ± 3.54, min = 18, max = 37) with self-reported fear of spiders, who were recruited from a database for study participation hosted by the University of Vienna. Exclusion criteria were pregnancy, past or present diagnosed psychiatric illnesses, or a history of drug or alcohol abuse. Inclusion criteria were eligibility to undergo an MRI and a “Spinnenangst” (i.e., “spider fear”) screening (SAS) [29] sum score ≥ 8 [42]. The study was approved by the Ethics Committee of the University of Vienna (IRB numbers: 00584, 00657). Participants provided written informed consent. The recruitment period for the study lasted from July 15, 2021, to January 23, 2023. The study was conducted in German. In the present article, we analyzed the data of a subset of these participants, who completed at least the initial session of the cBAT, resulting in a sample of n = 47 participants (39 female, 8 male; mean age = 22.53, SD = 3.57, min = 18, max = 37). The sample had a mean FSQ sum score [29] of 55.77 (min = 9, max = 90, SD = 20.84), which reflects at least moderate levels of spider fear across the participants [43].

### Measures

The following self-report questionnaires were filled out by the participants. For each questionnaire we provide a short description together with the Cronbach’s alpha (α) as stated in the respective publication: (1) The German version of the Fear of Spiders Questionnaire (FSQ) [29], which consists of 18 question items related to fear of spiders (e.g., *“If I came across a spider now, I would leave the room.”)* [27] that participants have to answer on a 7-point Likert scale (α = 0.96) [29]; (2) the German adaptation of the Spider Phobia Questionnaire (SPQ) [29], which is a 43-item self-report tool where participants answer “yes or no” questions, indicating their subjective level of fear (α = 0.84); (3) the German “Spinnenangst” Screening (SAS) [29], which is a 4-item questionnaire on fear of spiders, employing a 7-point Likert scale that was introduced as a screening tool for fear of spiders (α = 0.92); (4) the German State-Trait Anxiety Inventory (STAI) [44], composed of two scales (state-anxiety and trait-anxiety, respectively), each of which consists of 20 items that are answered on a 4-point scale (α = 0.90); (5) a German disgust propensity questionnaire (FEE) [45], consisting of 37 items that are answered on a 5-point scale, indicating disgust propensity (α = 0.90); and (6) a German disgust sensitivity questionnaire (SEE) [46], employing 7 items and a 5-point scale to answer questions related to disgust-sensitivity (α = 0.85).

Moreover, the novel cBAT [41] was employed, where participants were asked to unblur three highly blurred images of spiders up to a point where they still felt comfortable viewing them. The cBAT was programmed in PsychoPy, based on a script that was adapted from the original work of Peirce et al. [47], and completed by the participants on a university laptop computer. The following instructions were displayed on the screen: *“On the following pages, you will see blurred, unrecognizable images of spiders. Use the slider to sharpen each image to the point where you still feel comfortable looking at it. Important: Keep the left mouse button pressed while moving the slider. As soon as you release the left mouse button, your answer will be saved. We will start with a practice round with a neutral image. This will be followed by the spider images. If you want to start, press the Space key*.”(translated from German) [41]. The unblurring scale was positioned below the image, labelled with “min” and “max”, and the slider was continuously adjustable. Sharpening of the images occurred in a logarithmic manner, with values from 1 to 101 stored on a linear scale. The images displayed in the cBAT showed relatively large spiders (all with visible body hair), one of which was standing on the palm of a human hand, another one that was directly facing the camera, and another one that was visible from the top. The order of the images was randomized. In our previous work [48], these three images were rated according to fear on a scale from 0 to 100 by spider-fearful adults with an FSQ sum score of ≥ 24 [43], resulting in high mean fear ratings of 69.739, 70.575, and 80.138, respectively. The three trials of the cBAT resulted in a mean unblur value per participant (i.e. cBAT score). The more the images were unblurred on average, the higher the cBAT score. Moreover, we measured the total time participants needed to complete the three trials of the cBAT (i.e. cBAT decision time), after reading the instructions and completing the practice trial. In the initial session, the average cBAT decision time was approximately 30 seconds (mean = 30.95, median = 29.22, min = 10.30, max = 63.45, SD = 12.84).

Besides the cBAT, participants completed a rl-BAT, where they were asked to approach a real huntsman spider (*Heteropoda ssp*.; Latreille, 1804) in a terrarium. Participants received the following instructions from the experimenter before entering the room with the terrarium: *“In this room, we have a terrarium containing a spider. We would like to ask you to approach the terrarium up until a point where you still feel comfortable, and then stop and let us know. If you want to go all the way to the terrarium and feel comfortable, you may open the transparent lid, but you don’t have to do* this.” (translated from German) [41]. Thus, unlike the BAT by Öst et al. [9], our BAT’s final step did not involve any physical contact with the spider. In our rl-BAT, the spider was inanimate and mounted in a natural-looking position, so that participants were unaware of its inanimate state. In a post-study feedback questionnaire only one out of all participants in the original study by Zhang et al. [41] reported doubts regarding the authenticity of the spider. The distance that participants walked towards the terrarium (i.e., rl-BAT approach distance) was measured in meters using a laser distance meter. Whether participants first completed the rl-BAT or the cBAT was counterbalanced.

### Procedure

At a point between one and 14 days before the initial cBAT session, participants completed an online survey which consisted of the self-report questionnaires listed above. The survey was completed online on the University of Vienna’s SoSci platform [49]. On the day of the initial session, participants completed both the cBAT, as well as the rl-BAT at the University of Vienna. The cBAT and the rl-BAT took place in different rooms. Subsequently, participants underwent a functional magnetic resonance imaging (fMRI) scan, during which they were presented with 225 images of spiders and spider-related content. Between one and three days after the fMRI scan, participants rated the images they had been presented with according to fear, disgust, and approach-avoidance, which was done from home. Between four and five weeks after the initial BATs, participants returned to the University of Vienna for a follow-up, in which they completed the FSQ, as well as the cBAT and rl-BAT. The follow-up served the purpose of examining a potential reduction in fear due to the exposure to spider stimuli, which were presented to the participants during the fMRI session in the neuroimaging study [41]. Fourty-four out of the 47 initial cBAT completers participated in the follow-up.

### Statistical analysis

We employed Spearman correlations to assess the cBAT’s validity and to test our hypotheses. Firstly, we assessed concurrent validity (i.e., correlation with an established measure of the construct, obtained at the same time) [38,50] by correlations between rl-BAT approach distances and cBAT scores. Secondly, to test convergent validity, we correlated the initial cBAT scores with FSQ, SPQ and SAS sum scores, respectively. To enable a comparison with the correlational results of two already established measures, we also correlated rl-BAT approach distance with FSQ sum scores. Thirdly, to examine divergent validity (i.e., relations with measures of dissimilar constructs and is also called discriminant validity) [38], correlational tests with the STAI were performed. To test for a relationship between decision time and fear of spiders, we correlated the total time needed to complete the three cBAT trials (i.e., “cBAT decision time”) with rl-BAT approach distance, as well as with FSQ sum scores. As the experimenter was present in the room with a subset of the participants during the cBAT [41], which might have had an impact on participant’s responses, we also carried out a sensitivity analysis in the following manner: Firstly, as an indicative control check whose results should be interpreted with caution given the sample size, we employed a Mann-Whitney U test to examine a potential difference in the cBAT scores of participants where the experimenter was absent (n = 41), and those where the experimenter was present (n = 6) in the initial session. This was not tested at follow-up as the experimenter was present in the room with only three participants (out of the subsample analyzed in this study) in this session. Secondly, we used the subsample of participants where the experimenter was absent (n = 41 in the initial session, n = 40 at follow-up) to recompute the correlational tests relevant to our hypotheses and validity tests. As approximately one quarter of the participants only filled out the follow-up FSQ after completing the cBAT and the rl-BAT [41], we only used initial FSQ scores for the present analysis to avoid potential confounds due to the reversed sequence. Consequently, we employed cBAT, rl-BAT and questionnaire data for the analysis of the initial session, while only cBAT and rl-BAT data, but no questionnaire data was used in the follow-up analysis. Holm-Bonferroni corrections (p_adj_) were applied to correlational tests (within the initial data as well as within the follow-up data) to account for multiple comparisons [51,52]. The analysis was performed in R [53] using RStudio [54].

## Results

### Concurrent and convergent validity

**Fig 1:**
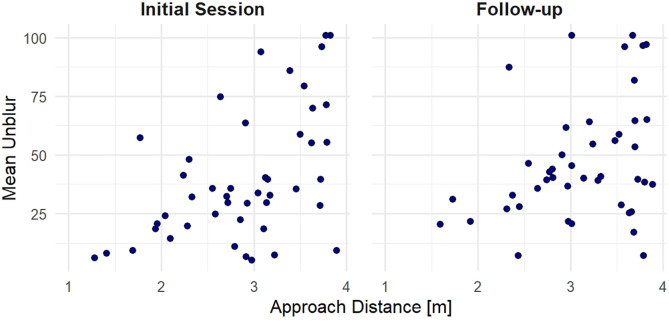
Scatter plots illustrating the relationship between approach distance (in meters) during the real-life BAT, and mean unblur scores during the computerized BAT in two experimental sessions. “Initial Session” (left) and “Follow-up” (right). In the initial session, a Spearman correlation showed a significant, moderate positive relationship, while no significant relationship was observed in the follow-up session after Holm-Bonferroni correction was applied.

**Fig 2:**
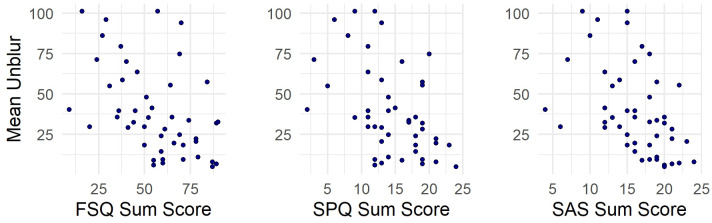
Scatter plots showing the relationship between the initial mean unblur scores of the computerized BAT and three different sum scores of self-report questionnaires measuring spider fear. FSQ (left), SPQ (center), and SAS (right). Spearman correlation tests revealed significant, moderate negative relationships between the sum scores for all three questionnaires.

The correlational analysis showed a positive relationship between cBAT scores and rl-BAT approach distances in the initial session, r_s_(47) = 0.48, p < 0.001, p_adj_ = 0.009, indicating moderate concurrent validity. At follow-up, this correlation was no longer significant after Holm-Bonferroni correction was applied, r_s_(44) = 0.32, p = 0.034, p_adj_ = 0.137. Regarding convergent validity, the analysis revealed moderate negative correlations between initial cBAT scores and FSQ sum scores, r_s_(47) = −0.51, p < 0.001, p_adj_ = 0.003, SPQ sum scores, r_s_(47) = −0.48, p < 0.001, p_adj_ = 0.008, and SAS sum scores, r_s_(47) = −0.55, p < 0.001, p_adj_ = 0.001, indicating that individuals with higher sum scores unblurred the images less than those with lower sum scores. Correlations related to concurrent and convergent validity are illustrated in Figs 1 and 2, respectively. Moreover, we found a moderate negative correlation between the initial rl-BAT approach distance and FSQ scores, r_s_(47) = −0.45, p = 0.002, p_adj_ = 0.019.

### Divergent validity and decision time

Correlations of the initial cBAT scores with constructs different from spider fear were non-significant for state anxiety, r_s_(47) = 0.01, p = 0.952, p_adj_ = 1, and trait anxiety, r_s_(47) = −0.33, p = 0.026, p_adj_ = 0.206. Regarding cBAT decision time, there were no significant correlations with rl-BAT approach distance (initial session: r_s_(47) = 0.18, p = 0.234, p_adj_ = 1; follow-up: r_s_(44) = 0.02, p = 0.880, p_adj_ = 1), nor with FSQ sum scores, r_s_(47) = 0.03, p = 0.86, p_adj_ = 1.

### Sensitivity analysis

A Mann-Whitney U test revealed no significant difference between participants where the experimenter was present in the room during the cBAT and participants where the experimenter was absent in the initial session, U(6, 41) = 0.96, p = 0.352. Regarding tests for concurrent and convergent validity, the correlational analysis showed the same patterns as observed in the primary analysis with slightly varying correlation coefficients. Like in the full sample, cBAT scores were moderately correlated with rl-BAT approach distance in the initial session, r_s_(41) = 0.44, p = 0.004, p_adj_ = 0.038, whereas this effect was no longer significant at follow-up, r_s_(40) = 0.30, p = 0.062, p_adj_ = 0.185. Moreover, initial cBAT scores were moderately correlated with the FSQ, r_s_(41) = −0.51, p < 0.001, p_adj_ = 0.009, SPQ, r_s_(41) = −0.50, p < 0.001, p_adj_ = 0.009, and SAS, r_s_(41) = −0.55, p < 0.001, p_adj_ = 0.003. There were no significant correlations with state-anxiety (r_s_(41) = −0.05, p = 0.712, p_adj_ = 1) and trait-anxiety (r_s_(41) = −0.33, p = 0.037, p_adj_ = 0.261). Like in the full sample, cBAT decision time was not correlated with rl-BAT approach distance (initial session: r_s_(41) = 0.15, p = 0.363, p_adj_ = 1; follow-up: r_s_(40) = −0.01, p = 0.937, p_adj_ = 1), nor with FSQ sum scores (r_s_(41) = 0.01, p = 0.949, p_adj_ = 1).

## Discussion

In the present study, we assessed the validity of a novel computerized behavioral avoidance test [41] for measuring fear of spiders compared to a conventional real-life behavioral avoidance test and self-report questionnaires. This cBAT was designed for ease of use, quick administration and requires minimal instructions for participants and no supervision from an experimenter, making it readily incorporable in online apps or devices. Our findings indicated that the cBAT demonstrated moderate concurrent validity with rl-BAT approach distances and moderate convergent validity with self-report measures of spider fear. However, the correlation between the cBAT and rl-BAT was no longer observed at follow-up, suggesting that while the cBAT may have potential for initial assessments, it may require further refinement to enhance its robustness and reliability.

We hypothesized that cBAT scores would be moderately to strongly correlated with established measures for fear of spiders and found moderate correlations with rl-BAT approach distance, as well as with self-report questionnaire sum scores measuring spider-fear in the initial session. These results indicate moderate concurrent and convergent validity, respectively. However, concurrent validity did not persist at follow-up, which suggests limitations in the measure’s robustness. As a reduced, image-based task, the psychological mechanisms involved in the cBAT may differ substantially from real-life settings, and the task might be subject to more noise and variation and be less sensitive than established tools. While the task might involve similar psychological components in terms of avoiding a fear-inducing stimulus, its setting is substantially less immersive, which is relevant when for comparisons with similar measures, as well as with regard to the task’s ecological validity. Additionally, while our analysis only revealed moderate correlations, Grill et al. [40], who also developed a computerized BAT, found that the correlations with FSQ scores and with a rl-BAT were strong, with correlation coefficients of −0.76, and 0.87, respectively. A potential reason for this could be the design of the participant task. Different to our cBAT, where participants unblurred three images of spiders, the task design Grill et al. [40] follows the same steps that are completed in a rl-BAT (i.e., approaching a spider). Thus, stronger correlations between the measures can be expected. Also, our rl-BAT was distinct from the one used by Grill et al. [40]. We measured the participants’ distance to a terrarium on a metric scale using a laser distance meter, while Grill et al. [40] asked participants to complete a series of different steps as described above. Interestingly, correlations of our rl-BAT with FSQ scores were only moderate, and notably less pronounced than in the study by Grill et al. [40]. The results of two other studies are relevant with regard to this matter: 1) A computer-delivered BAT by Meng et al. [37], which is more comparable to the cBAT assessed in the present study, also demonstrated strong correlations with established measures, and (2) another digital BAT with a relatively simple task, where participants indicated approach distance through a mouse click [17], showed only weak to moderate correlations with the FSQ. While BATs and the FSQ aim for the same theoretical construct, their operationalization is different. Moreover, various versions of rl-BATs, for example by Öst et al. [9] and Cochrane et al. [32], as well as computerized BATs, for example by Dibbets and Schruers [17] and Meng et al. [37], have been developed, each potentially capturing different aspects of spider-related avoidance behavior. Given the results of the present study, one can argue that our task might not capture the full complexity of such behavior. However, spider phobia consists of different, interrelated psychological constructs, particularly fear and disgust, which are strong and aversive emotions [55]. Avoidance behavior goes hand in hand with these emotions [43]. Considering the interrelated nature of the constructs that constitute spider phobia, it remains a challenge to accurately assess fear of spiders.

As hypothesized, we found that cBAT scores were not significantly correlated with measures of constructs different to fear of spiders. Specifically, our analysis showed no relationships with state-anxiety and trait anxiety. Thus, the cBAT demonstrated some divergent validity. However, it must be mentioned that these constructs were assessed within the scope of a study on fear of spiders. Consequently, these results should be viewed with caution. An assessment of divergent validity with other measures would have been preferable and should be carried out in future studies. Contrary to our hypothesis, the total time needed to unblur the images in the cBAT was neither associated with rl-BAT approach distance, nor with FSQ sum scores. Therefore, unlike in the work of Binder et al. [34] and Frumento et al. [35], the time needed to perform the task was no reliable indicator of fear of spiders. Nevertheless, this is in line with the results of Meng et al. [37], who also found that decision time in their computer-delivered BAT did not allow them to differentiate between levels of spider fear. Hence, it remains an open question whether decision time is a crucial factor allowing to discriminate between more and less spider-fearful individuals.

The present study has several limitations. Firstly, as our previous work [41] did not require a control group, we were unable to assess the baseline variation of our measure in the population without spider fear and we could not test if our measure differentiates between non- and fearful individuals. However, our sample ranged from individuals with very low to high fear of spiders. Secondly, as participants did not fill out scales measuring constructs that were entirely unrelated to fear of spiders, divergent validity could only be analyzed with the measures that were available. Thirdly, no clinical assessment was provided, and we solely relied on self-report questionnaires and rl-BAT data as measures of fear of spiders. Finally, the experimenter stayed in the room with the participants during the cBAT on a small number of occasions, which may have influenced participants’ responses. Regarding this matter, we conducted a sensitivity analysis, where the respective participants were excluded, and overall found the same patterns as in the full sample, which strengthens the findings reported in the analysis. Nevertheless, the generalizability of our results is limited by the fact that the participants were recruited via a university database for study participation, with the majority of the sample consisting of young adults (mostly females), covering a relatively small age range. Future studies could consider the limitations listed above and examine the cBAT in clinically diagnosed individuals, using a healthy control group and different additional measures. Moreover, the cBAT could be refined by adding some complexity to the participant task to better capture the different aspects of avoidance behavior and make it more ecologically valid. For instance, like in the study by Grill et al. [40], it might be beneficial to display a hand that can be controlled by participants.

In light of the results of the present study, we conclude that the cBAT is a limited measure for fear of spiders. Importantly, the cBAT might not fully capture the complexity of spider-related avoidance behavior compared to tasks that simulate real-life scenarios more closely. Nonetheless, this study has the potential to inform the design of comparable tasks, specifically regarding the importance of balancing accessibility and ease of use with complexity and sensitivity. Notably, spider phobia is constituted by different constructs (i.e., fear, disgust, and avoidance) [43,55], resulting in complex psychological underpinnings, particularly as these constructs are interrelated [55,56]. More investigations are warranted to determine the task design and complexity that is needed to accurately measure and reflect avoidance behavior in fear of spiders. Given the need for a quick assessment of fear of spiders [36], it is worth developing and investigating computerized measures with straightforward experimental tasks. Such designs could be used in classical ET as well as virtual reality ET and would be especially beneficial for novel online treatment applications that can be accessed via the internet and do not require extra equipment. While the cBAT assessed in the present study comes with these advantages and can be applied with minimal instructions and in a short amount of time, the current design appears to be suboptimal and might not fully capture the complexity of avoidance behavior. We propose further refinement or the use of alternative measures that can reliably be used as pre- and post-treatment assessments in exposure therapy for fear of spiders.
